# The potential of a “next generation” of community health workers to accelerate progress in reaching Universal Health Coverage and Health for All

**DOI:** 10.1371/journal.pgph.0007545

**Published:** 2026-10-30

**Authors:** Henry B. Perry, Abhay Bang, Steve Hodgins, Madeleine Ballard, Jeffrey D. Sachs, Abnet Z. Muluneh, Mushtaque Chowdhury, Mark Mwenda, Raj Panjabi

**Affiliations:** 1 Johns Hopkins University, Baltimore, Maryland, United States of America; 2 Society for Education, Action, and Research (SEARCH), Gadchiroli, Maharashtra, India; 3 University of Alberta, Edmonton, Alberta, Canada; 4 Community Health Impact Coalition (CHIC), London, United Kingdom; 5 Icahn School of Medicine at Mount Sinai, New York, New York, United States of America; 6 Columbia University, New York, New York, United States of America; 7 International Institute for Primary Health Care – Ethiopia, Addis Ababa, Ethiopia; 8 BRAC University, Dhaka, Bangladesh; 9 County Government, Kiambu, Kenya; 10 Division of Global Health Equity, Department of Medicine, Mass General Brigham and Harvard Medical School, Boston, Massachusetts, United States of America; 11 Flagship Pioneering, Cambridge, Massachusetts, United States of America; Johns Hopkins School of Medicine: The Johns Hopkins University School of Medicine, UNITED STATES OF AMERICA

## Abstract

Many health systems, particularly in low- and lower middle-income countries (LMICs), are lagging in their progress in achieving Universal Health Coverage and delivering on the promise of Health for All, constrained by workforce shortages, fiscal ceilings, and inadequate outreach to those lacking basic and essential services. Stark inequities in coverage of services and in health status persist. Strengthening community health worker (CHW) programs provides the fastest and most cost-effective route for health systems to achieve their full potential. CHWs should become the backbone of primary health care (PHC) and the foundation of health systems. Evidence from more than a decade of national-scale experience demonstrates that CHWs who are appropriately trained, paid, equipped, and supervised can extend high-quality essential services to the last mile while improving the overall efficiency of the health system. We are at a point in time when donor strategies and private investment are captivated by the promise of digital health and artificial intelligence. The most reliable approach to saving more lives is still a paid, supported CHW empowered with digital tools. At the same time, the contraction of Official Development Assistance (ODA) creates both risk and opportunity. ODA must pivot from supporting services to catalytic capital: time-bound, matching investments that accelerate governments’ own proCHW policy. A “next generation” of CHW programs in LMICs would enable CHWs to visit every home monthly and provide a broad array of PHC services as a result of adequate remuneration and appropriate training, supervision, logistical support, and integration into the health system. This could yield a highly favorable economic return—through productivity gains from better health, reduced health-care expenditures, and pandemic-risk mitigation. A “next generation” of CHW programs is our best bet for accelerating progress in achieving Universal Health Coverage and Health for All sooner rather than later.

## Introduction

The desire – and right – to live a long, healthy, and productive life is one the most fundamental of human hopes and aspirations. It is one of the principal goals that societies and their governments strive to fulfil. The Universal Declaration of Human Rights, which is the United Nations’ moral charter and whose 75^th^ anniversary was celebrated in 2023, resoundingly affirms that everyone has the right to accessible and effective basic health care services. As enshrined in the Declaration of Alma-Ata, arising from the International Conference on Primary Health Care in 1978 [1], the achievement of Health for All requires the ready availability of “essential health care based on practical, scientifically sound and socially acceptable methods and technology made universally accessible to individuals and families in the community through their full participation and at a cost that the community and country can afford to maintain at every stage of their development in the spirit of self-reliance and self-determination” [1].

Who could have imagined in the mid-20^th^ century, when the Universal Declaration of Human Rights was created and the United Nations system was formed following a global depression and two world wars, that three-quarters of a century later we would find ourselves living in a world in which 4.5 billion people – more than one-half of the world’s population – still lack access to many essential health services [2]. And furthermore, according to the Global Health Workforce Alliance of the World Health Organization (WHO), 1 billion people have never seen a health care provider [3]. One-third of the 34 million births occurring each year in low-income countries still take place at home without the presence of a suitably skilled and equipped healthcare provider [4]. As shown in Box 1, more than 25 million people are dying every year in low- and middle-income countries (LMICs) from readily preventable or treatable conditions, and millions more are suffering the consequences of undernutrition, lack of readily accessible family planning services, and readily treatable visual problems, among others.

**Box 1. Current numbers and causes of readily preventable mortality and serious morbidity globally annually.**

- 1.9 million stillbirths [4]
- 2.3 million neonatal deaths [4]
- 2.7 million deaths of children 1–59 months of age [4]
- 0.3 million maternal deaths [4]
- 200 million undernourished children (22% of children younger than 5 years of age are stunted, with low height for age, a marker of chronic undernutrition; 7% are wasted, with low weight relative to their height, a marker of acute undernutrition [5])
- More than 250,000 deaths in children due to stunting and more than 1 million deaths in children due to wasting (often as a result of their increased susceptibility to infection [6]; undernourished children also have worse cognitive development [7] and lower earnings as adults [8]
- 257 million women with an unmet need for family planning [9]
- 1.2 million deaths from tuberculosis [10]
- 0.6 million deaths from HIV/AIDS [11]
- 0.6 million deaths from malaria [12]
- 10 million deaths from complications of hypertension [13]
- 1 billion people with readily treatable vision impairment (including 826 million with presbyopia, treatable with reading glasses, and 94 million with treatable cataracts) [14]
- Over 5 billion people lack access to safe surgical services, and 4.7 million deaths annually are attributed to untreated surgical conditions, accounting for roughly 10% of all global deaths, and 93% of people in LMICs lack access to basic emergency surgical services [15]

Now, already past the halfway point between their establishment in 2015 and the date for achieving them (2030), the Sustainable Development Goals (SDGs) are in deep trouble: only one-sixth of the 140 targets are on track to be achieved [16]. Among the health-related targets, fewer than one-tenth are on track, two-thirds are showing some but insufficient progress, one-fifth have stagnated or regressed, and data are missing for the others [16]. Although the coronavirus pandemic that began in 2019 (COVID-19) and the global economic downturns it produced are certainly partly to blame, the more important contributor for slow progress on the health-related SDGs has been inadequate investments in community health workers (CHWs) and community-based primary health care (CBPHC).

Here we highlight the potential of CHWs to reverse the stagnation in achieving Universal Health Coverage and reaching Health for All at this inflection point. Emerging digital tools (artificial intelligence, decision-support tools, registries, advisory dashboards, and supply chain monitoring) [17–19] and strengthened PHC facilities [20] will no doubt be helpful, but relying on them alone without a “next generation” CHW workforce in frequent personal contact with all households will lead to further disappointments. And when these CHWs are empowered by digital and artificial intelligence tools, we can expect to see remarkable improvements in health system effectiveness and quality of care.

## The effectiveness of community health workers in providing community-based primary health care

CBPHC refers to the provision of basic and essential promotive, preventive, curative and rehabilitative services in the community outside of health facilities and in the home by CHWs who are most effective when they work as part of a health team connected with health facility services. CBPHC also includes the engagement of communities in the planning, implementation and assessment of these services.

CHWs make possible the desirable but often missing attribute of “community engagement” in health care. Through their lived experience in the community, CHWs know about the health needs and priorities of the people. Their involvement in local planning and priority setting makes health care more need-based.

For more than a century there have been efforts to extend the benefits of modern medicine and public health to communities that had no access to modern medical care by providing local people with short-term, on-site training. These efforts first began in the 1920s in China with illiterate, so-called “barefoot doctors” who received three months of training [21]. Then, in the 1960s and 1970s similar programs were introduced throughout the world. Early seminal publications that recognized and promoted the importance of CHWs included, among others, Newell’s 1975 *Health by the People* [22], the International Conference on Primary Health Care’s 1978 Declaration of Alma-Ata [1], and Werner’s prescient “Village Health Worker: Lackey or Liberator?” [23].

From 1962 to 1982, the life expectancy in China nearly doubled (from 35 to 60 years); the “barefoot doctors,” whose numbers rose to more than one million during this period, were an important contribution to this remarkable achievement [24–27]. Now, more than four decades later, the evidence base for how population health can be improved through CBPHC has mushroomed. Exemplary national CHW programs now exist in Bangladesh (Box 2), Brazil, Costa Rica, Ethiopia (Box 3), India (Box 4), Liberia, Nepal, Rwanda and Zambia, among others [28]. Even in the United States CHW programs are proliferating although so far they are small-scale programs [29].

**Box 2. Bangladesh, the quintessential national example of the power of CHWs [30].**

At the time of independence in 1971, Bangladesh was the second poorest country in the world (after Burkina Faso), with one-quarter of its children dying before reaching age 5 (and 25% of these deaths from diarrhea), an immunization coverage rate of 2%, and a total fertility rate of 6.6 births per woman. Now, a half-century later, only 3% of children are dying over the first 5 years of life. Bangladesh has the highest usage rate in the world of oral rehydration therapy for children with diarrhea (87%). Child immunization coverage is nearly 100%, the total fertility rate is below replacement level, 70% of cases of pulmonary-active TB are detected and treated, and 90% of TB cases complete their treatment. These results could not have been achieved without CHWs making regular home visits.

**Box 3. Ethiopia’s Health Extension Program [32].**

In 2003, the Ethiopian government committed itself to providing one year of training to female Health Extension Workers (HEWs), each of whom would serve 2,500 people by providing care at a village health post and also by visiting every home. They received a government salary with benefits. Over the following 10 years, the program deployed 34,000 HEWs who were stationed in pairs at a health post serving 5,000 people. There are now more than 40,000 HEWs working. Helping them are 3 million Women’s Development Army volunteers (one for every 5–6 households). The coverage of basic and essential services increased rapidly. Ethiopia was one of only a few African countries to reach the 2015 Millennium Development Goal for Child Health (reduce the country’s under-5 mortality rate by two-thirds between 1990 and 2015). In 15 years, the contraceptive prevalence rate increased from 8% to 35%, an extraordinary rate of increase, especially for Africa.

**Box 4. India’s Accredited Social Health Activist (ASHA) Program, the world’s largest national example of the feasibility and uses of CHWs [31].**

In the early 2000s, the Government of India established the National Rural Health Mission to upgrade its rural PHC system. At that time, three programs had demonstrated that female CHWs with minimal formal education could bring about notable improvements in rural health conditions provided that they had strong training and support. These three programs were the CHW programs of Comprehensive Rural Health Project in Jamkhed, Maharashtra (1970-present); SEARCH (Society for Education, Action, and Research in Community Health) in Gadchiroli, Maharashtra (1980-present); and the CHW program operated by the state of Chhattisgarh (2003-present). ASHA means “hope” in Hindi. Beginning in 2005, ASHAs were deployed in high-priority rural areas. The program gradually expanded to other rural areas of India and in 2013 it expanded to serve urban areas as well. There are now more than 1 million ASHAs, making it the largest CHW program in the world. Each ASHA serves approximately 1,000 people with health-related education and counseling, assisting with the utilization of health services (notably immunizations, family planning, birthing care at health centers, and home-based newborn care), and engaging the community in collective action through facilitating village health and nutrition committees. Their role is now expanding to include detection and treatment of non-communicable diseases such as hypertension and diabetes.

The WHO has published the most extensive review at present of the world’s experience with community health programs – not only in LMICs but in high-income countries as well [33]. WHO guided the collaborative production of evidence-based guidelines for CHW programs [34], and recommended that CHWs be recognized as an integral part of the health workforce throughout the world where unmet health needs persist (which is, in fact, almost everywhere in the world).

Rather than considering CHWs as a second-class solution to a temporary problem that will gradually fade away as facility-based services strengthen, strong CHW programs have the potential to be the vanguard of accelerating progress in achieving Universal Health Coverage and Health for All, even in high-income countries. CHWs can play an important role in addressing epidemiological priorities. If the known evidence-based child survival interventions that can be provided in the community reached 90% of those who needed them, the mortality rate of children in priority countries could be reduced by one-third, saving millions of lives each year [35]. If one adds the benefits from reaching similarly high levels of coverage for family planning services, HIV screening and treatment, TB screening and treatment, as well as screening and treatment for hypertension and diabetes, the number of lives saved would be considerably greater.

Even in the United States, CHWs are improving the management of chronic conditions, increasing access to preventive care, improving patients’ experience with care, reducing health care costs, and advancing health equity [29]. In 2024, the United States Centers for Medicare and Medicaid Services (CMS) began reimbursing for CHW services.

## Why are community health workers such a powerful resource for strengthening health systems?

By regularly visiting every household, the services that CHWs can provide are equitable, meaning that the more disadvantaged segment of the population receives at least the same coverage of services as the more advantaged segments. In many cases, CHW services are pro-equitable, meaning that the most disadvantaged receive higher priority and achieve higher coverage [36]. When CHWs carry out routine systematic home visitation (which many do now [28]), a much higher level of equitable coverage of service delivery and health impact can be achieved than is possible through facility-based service delivery alone. CHWs can also be highly effective when facilitating women’s groups [37,38].

CHWs, who are usually from the same community and share the lived experience and expertise of the people they serve, are able to relate in a culturally appropriate way in the local language. They are available 24/7. They are highly motivated, compassionate, and eager to help with problems that arise. They often even accompany their seriously ill patients to the hospital, many times at their own expense. Consequently, for their neighbors they become a trusted and vital link to the health system.

Beyond the direct health benefits of a strong CHW program, there are additional benefits that cannot be achieved with stronger facility-based programming alone. Elsewhere [39], we have projected the costs and estimated the economic benefits of a “next generation” CHW program for low-income and lower middle-income countries that broadens the services that CHWs provide and enables monthly visits to all homes. Such a “next generation” CHW program has the potential to achieve a highly favorable return on investment. This would be achieved by worker productivity gains as a result of lives saved and improved nutrition, reduced costs of care by shifting services to lower-level less expensive health care providers and by making services available closer to home, and by providing a stronger focus on prevention and early treatment.

CHWs can provide community-based surveillance for disease outbreaks and registration of vital events. And once an outbreak has been detected, CHWs are on the frontline helping communities control and mitigate the effects. CHWs often kept essential services running during the COVID-19 pandemic, often without or protective equipment [40].

When CHWs are paid (which they should be, particularly if they routinely work more than 8 hours a week), the income provides benefits for their families’ health and for the education of their children (especially when the CHW is a woman). It also provides a small but significant boost to the local economy. And, for women CHWs (including those who are not paid), they become more self-confident and empowered to address gender disparities and other restricting local norms. Additional benefits accrue with employing young people as CHWs, providing a much needed source of employment where youth unemployment is high.

CBPHC can make an important contribution responding to the current global epidemiological transition to non-communicable diseases as the major disease burden. Home-based screening for hypertension, diabetes, and mental illness, task-sharing for treatment of these diseases, and linking to higher levels of care when needed provide the opportunity to build a much stronger health system than would be possible without CBPHC.

Hypertension is now the leading cause of preventable deaths worldwide [41]. It is the most important readily modifiable risk factor for heart disease, kidney failure, and stroke. In LMICs, 32% of the adult population is hypertensive, and only 8% of those with hypertension have their blood pressure controlled [42]. A recent randomized controlled trial from China demonstrated that 57% of patients treaated by CHWs (called village health doctors there) had a normal blood pressure compared to 20% in the control group [43]. By routinely screening for hypertension, promoting appropriate dietary and lifestyle modifications, providing treatment under a supervised protocol, and monitoring medication adherence, CHWs could lead the way in achieving better population outcomes for this global health priority. The potential contribution of CHWs to the control of other non-communicable diseases is also promising [44].

Aside from the increasing global importance of non-communicable diseases, two other issues of growing global importance for which a “next generation” CHW program could make an important contribution are climate change and humanitarian crises. Increasing temperatures, extreme weather events, natural disasters and political conflict leading to disruptions in the daily lives of people, including migration, are all likely to become more common and more problematic. A “next generation” CHW program would help communities to be more resilient in responding to these challenges through their ability to mobilize community-led responses and maintain essential services, as they so admirably did during the COVID-19 crisis.

We are at a potential inflection point in global health. Health systems—especially in low-income countries—are failing to deliver on the goal of Universal Health Coverage and the promise of Health for All. They are constrained by workforce shortages, fiscal ceilings, and inadequate outreach to those lacking basic and essential services. Stark inequities in coverage of health services and in health status persist. At the same time, the entire landscape for global health is undergoing radical change as donors make major cutbacks in their support. We now have the opportunity to re-envision how to do more with less, and how to help health systems become more effective, more efficient, and more equitable at the same time. The best way to do this is readily available and affordable: through strengthening CBPHC and building strong CHW programs. Progress has been constrained by lack of policy prioritization, competing fiscal pressures, and challenges in sustaining long-term investments in CHWs. Creating a “next generation” of CHWs who make up a well-trained and equipped workforce that reaches every household every month can be done more quickly and at a lower cost than any other alternative.

Well-trained, salaried, appropriately supported, and well-supervised CHWs provide the best opportunity for getting health systems on a faster track toward Universal Health Coverage and Health for All. The only other alternative – expanding the number of facility-based PHC services provided by higher-level health workers *without* the community outreach services that CHWs provide – is a costlier and slower path toward this goal that will likely fail to make a major dent in health inequities.

There now finally is a widespread recognition of the need to strengthen CHW programs, especially in Africa. Two-thirds of the countries of Africa now recognize CHWs as part of the national health workforce, and one-half of countries there pay CHWs through their national wage system [45]. Plans are underway, led by the African Union, to expand the CHW workforce in Africa to 2 million as a continental policy [45]. Looking ahead into the next decade and beyond, we envision an exciting opportunity to build a “next generation” of CHWs who can play an even stronger role and in fact become the backbone of PHC.

Admittedly, the published literature is replete with examples of CHW programs where the performance has been less than hoped for [46,47]. But it is also true that CHW programs cannot reach their full potential unless CHWs are appropriately trained, supervised, and supported with the supplies and medicines they need. And, if they are working more than 8 hours a week, they should be appropriately remunerated.

## How would a “next generation” community health worker differ from the “current generation” community health worker?

At present there is a broad diversity of CHW programs throughout the world [28]. One could imagine a rich diversity of “next generation” CHW programs in the future, given the rich diversity of contexts in which these programs will be implemented. However, there are certain basic features that in our view such a “next generation” CHW program should have [34]. First of all, the job responsibilities and the catchment areas should be structured in such a way so that the workload is manageable. CHWs should have a broad set of skills and competencies to enable them to address the local epidemiological priorities, manage uncomplicated medical problems that arise, and refer patients with problems beyond their capabilities to manage. Second, there should be relatively frequent contact of CHWs with every household (preferably monthly), and more frequent contact with those households that have greater health needs. Third, all CHWs working more than 8 hours per week should be appropriately remunerated depending on the local wage scale, but volunteers spending less than 8 hours per week should not be excluded from participation. All CHWs should be appropriately trained and receive appropriate continuing education and supervision, and they should have career advancement opportunities. Broadly speaking, most CHW programs at present have a catchment area of 1,000 people or so for each full-time equivalent CHW [28]. In our view, a “next generation” CHW should have no more than 500 people (or 100 households) for which s/he is responsible.

Elsewhere [39], we have proposed and costed a generic “next generation” CHW program for low-income and lower-income countries that meet these criteria. According to our calculations, the financial return on investment for such a program over a 20-year period would be highly attractive. In our generic model, paid lower-level CHWs working one-third time would be responsible for 25 households and would visit each household monthly, and eight of them would be supported by a full-time CHW. Experimentation with different “next generation” CHW programs should be encouraged, with the most effective versions allowed to flourish.

In countries with existing CHW programs, CHWs could obtain additional training to transition into their new upgraded role, and senior CHWs could be employed as trainers of CHWs in this new program.

## What are the alternatives?

The standard government response to proposals for building strong CHW programs is that they are too expensive. The proper response is that governments cannot afford *not* to make the necessary investments if they are serious about making their health care system more efficient (by reducing costs by utilizing less expensive and more readily available sources of care) and more effective (by reaching those previously unreached by the health system). For the poorest countries, donor assistance will still be needed for some time. Strong and effective CHW programs are not cheap. And CHWs who work more than 8 hours per week should be paid a fair and just wage, as called for by the WHO and others [34]. Even strong CHW programs will have room for drawing on the spirit of volunteerism that exists within all communities that can enable volunteer CHWs working less than 8 hours per week to make important contributions [48].

Why not simply expand the number of PHC centers staffed by higher-level health workers and shut down CHW programs? This approach has a number of drawbacks. Posting higher-level health workers in the geographic areas of greatest need remains an enormous challenge in many countries. The geographic maldistribution of higher-level health workers in LMICs is well documented [49] and in many countries postings in more isolated areas remain unfilled.

Thus, it will be difficult to fill a major expansion in the number of posts for these positions even if the numbers of higher-level health workers increases substantially. CHWs, on the other hand, are already there, so the problem of “posting” them has already been solved. Secondly, constructing and equipping enough PHC centers that are readily accessible to the population will take much longer and cost much more than training and deploying CHWs. In order to achieve Universal Health Coverage with PHC facilities alone, these centers would need to be no more than 3–4 kilometers or a 45-minute walk away since utilization decreases exponentially beyond this point [50]. This is rarely the case at present.

The only viable alternative is not to depend solely on the expansion of PHC facilities but, instead, to build health teams that rely heavily on CHWs who are in regular contact with every household and who work in tandem with higher-level staff based at facilities. Such a system, as developed so well in Brazil [51], can reduce utilization of facilities.

## The role of donors

Historically, CHW programs have not been a priority for Official Development Assistance (ODA). Although in recent years donors have been giving more support, this funding has been primarily geared toward the community-based delivery of vertical interventions such as for immunization, child survival, family planning, or HIV and TB. In any case, donor support for these purposes historically has accounted for only 2.5% of development assistance for health [52]. And, of course, much of this is in peril due to major cutbacks in global funding – from $80 billion in 2021 to a forecast of $36 billion in 2030 [53]. The overwhelming majority of domestic government expenditures in LMICs has gone for services at higher-level PHC centers and hospitals. When health facilities provide services that save lives, everyone rejoices, of course. However, what is overlooked and uncounted is the number of persons who die because they did not or could not reach a health facility for appropriate treatment. Consequently, CHWs have too often been an “underfunded afterthought.” During the Ebola epidemic in Freetown, Sierra Leone, when all bodies had to be taken to a central site for burial, those involved told one of the authors (HP) that people were astounded by the number of infants and young children dying. In fact, very few of them were actually dying of Ebola.

Now is the time for donors to use their more limited funds to assist LMICs in finding ways to commit more domestic resources to build strong CHW programs. This means, for example, helping countries make the case for expanding domestic investments in CHW programming, thereby building stronger political will for CHW programs. It means assisting with the development and strengthening of national CHW associations. It also means assisting countries in finding new ways to fund CHW programs (such as through “sin taxes”). It means as well helping governments to find areas in their current health funding where inefficiencies exist and where funds could be freed up that could be used to support CHW programs.

As the Lancet Global Health Commission on Financing Primary Health Care has emphasized, we need transparency in how much governments are spending on PHC (and on CHW programs) relative to other elements of the health system, and we need to ensure that every locale receives its per capita share of resources required to provide PHC, especially those locales currently not well reached by existing health services [54]. ODA can be used to help countries achieve this – or at least help to make information about this publicly and widely available. Finally, ODA can be used to assist governments and civil society in building political support for better financing for PHC (and CHW programs), including ensuring that all locales receive their fair share of financing for PHC service [54].

## The way forward

Now is the time for a seismic shift in the structure of health systems – not only in resource-constrained settings but also in high-income settings. Even in high-income settings, evidence is growing rapidly about the effectiveness of CHWs in improving health outcomes, reducing health inequities, and generating a strong return on investment based on their ability to reduce health expenditures [29]. Based on their demonstrated effectiveness and their potential, appropriately trained and supported CHWs who are paid a just and fair wage and who have opportunities for career growth can lead the way toward achieving Universal Health Coverage and reaching Health for All sooner rather than later. By implementing the “next generation” CHW model, CHWs, with support from existing and emerging technology and with support from each level in the health system, would be able to make improvements in the health of populations that would not be achievable at the same cost by other means.

Support for strong CHW programs is now not only a moral imperative for health systems everywhere, but a wise investment for controlling health care costs. Three decades ago, Frankel wrote:

There is no longer any place for discussion of whether CHWs can be key actors in achieving adequate health care. The question is how to achieve their potential [55, p. 1].

The WHO now calls for CHWs to be recognized as an integral part, indeed the foundation, of effective health systems [34]. CHWs can be additional eyes and ears and even additional arms and legs of a health system. They can even expand the “brains” of the health system. National CHW associations, which are becoming active in many countries, provide CHWs with a voice at the table for negotiating their fair share of program support from the government.

The opportunity is available. The health and lives of billions of people depend on it. Less than 5% of the $2.1 trillion [56] that the world spends on armaments would be needed to provide the additional financial resources needed [57].

In 1972, one observer lamented, “If I were asked to compose an epitaph on medicine throughout the 20^th^ century, it would read: ‘Brilliant in its scientific discoveries, superb in its technological breakthroughs, but woefully inept in its application to those most in need’” [58]. Here we are now at the quarter-way point toward the end of the 21^st^ century. Is it not time to build “next generation” CHW programs that apply our scientific discoveries and technological breakthroughs to those most in need?

## Abbreviations

CBPHC: Community-based primary health care
CHWs: Community health workers
CMS: Centers for Medicare and Medicaid Services
COVID-19: Corona virus disease (2019 pandemic)
HEW: Health Extension Worker
HIV: Human immunodeficiency virus
ODA: Overseas development assistance
PHC: Primary health care
SDGs: Sustainable Development Goals (of the United Nations)
TB: Tuberculosis
WHO: World Health Organization

## References

[1] WHO, UNICEF. Declaration of Alma-Ata, 6-12 September 1978 Alma-Ata, USSR: World Health Organization and United Nations Childrens’ Fund; 1978. Available from: https://cdn.who.int/media/docs/default-source/documents/almaata-declaration-en.pdf?sfvrsn=7b3c2167_2

[2] WHO. Universal Health Coverage: Report by the Director-General. Geneva: World Health Organization; 2025. Available from: https://apps.who.int/gb/ebwha/pdf_files/EB156/B156_6-en.pdf

[3] GHWA. Imagine Geneva: Global Health Workforce Alliance. 2011. Available from: https://www.youtube.com/watch?v=tCSmIYmPOi4

[4] UNICEF. The state of the world’s children 2023: For every child, vaccination. Florence, Italy: United Nations Children’s Fund Global Office of Research and Foresight; 2023. https://www.unicef.org/reports/state-worlds-children-2023

[5] UNICEF, WHO, World Bank Group. Levels and Tends in Child Malnutrition. New York, NY, USA: UNICEF, World Health Organization, and World Bank Group; 2019. Available from: https://iris.who.int/bitstream/handle/10665/331097/WHO-NMH-NHD-19.20-eng.pdf?sequence=1

[6] GBD 2015 Risk Factors Collaborators. Global, regional, and national comparative risk assessment of 79 behavioural, environmental and occupational, and metabolic risks or clusters of risks, 1990-2015: a systematic analysis for the Global Burden of Disease Study 2015. Lancet. 2016;388(10053):1659–724. doi: 10.1016/S0140-6736(16)31679-8 27733284 PMC5388856

[7] Black RE, Victora CG, Walker SP, Bhutta ZA, Christian P, de Onis M, et al. Maternal and child undernutrition and overweight in low-income and middle-income countries. Lancet. 2013;382(9890):427–51. doi: 10.1016/S0140-6736(13)60937-X 23746772

[8] McGovern ME, Krishna A, Aguayo VM, Subramanian SV. A review of the evidence linking child stunting to economic outcomes. Int J Epidemiol. 2017;46(4):1171–91. doi: 10.1093/ije/dyx017 28379434 PMC5837457

[9] UNFPA. State of World Population 2022 -- Seeing the Unseen: The Case for Action in the Neglected Crisis of Unintended Pregnancy. New York, New York, USA: United Nations Fund for Population Activities; 2022.

[10] WHO. Global Tuberculosis Report 2025. Geneva: World Health Organization; 2025. Available from: https://www.who.int/teams/global-programme-on-tuberculosis-and-lung-health/tb-reports/global-tuberculosis-report-2025

[11] UNAIDS. Global HIV & AIDS statistics — Fact sheet. Geneva: UNAIDS; 2025. Available from: https://www.unaids.org/en/resources/fact-sheet

[12] WHO. Malaria - Fact Sheet. Geneva: World Health Organization; 2022. Available from: https://www.who.int/news-room/fact-sheets/detail/malaria

[13] WHO. Global report on hypertension 2025: High stakes – turning evidence into action. 2025. https://iris.who.int/server/api/core/bitstreams/93b8e571-00a2-4de3-bb34-30f99b384816/content

[14] WHO. Blindness and Vision Impairment: World Health Organization; 2023. Available from: https://www.who.int/news-room/fact-sheets/detail/blindness-and-visual-impairment

[15] Nepogodiev D, Ismail L, Meara JG, Roslani AC, Harrison EM, Bhangu A. Strengthening health systems through surgery. Lancet. 2024;403(10442):2358–60. doi: 10.1016/S0140-6736(24)01031-6 38782001

[16] UN. The Sustainable Development Goals Report: Special Edition. New York, NY, USA: United Nations; 2023. Available from: https://unstats.un.org/sdgs/report/2023/The-Sustainable-Development-Goals-Report-2023.pdf

[17] Kasper T, Yamey G, Dwyer S, McDade KK, Lidén J, Lüdemann C, et al. Rethinking how development assistance for health can catalyse progress on primary health care. Lancet. 2023;402(10418):2253–64. doi: 10.1016/S0140-6736(23)01813-5 37967568

[18] Craig A, Lawford H, Miller M, Chen-Cao L, Woods L, Liaw ST. Use of technology to support health care providers delivering care in low- and lower-middle-income countries: Systematic umbrella review. J Med Internet Res. 2025;27:e66288. 10.2196/66288 PMC12223456 40533075

[19] Srivastava D, Van Kessel R, Delgrange M, Cherla A, Sood H, Mossialos E. A framework for digital health policy: Insights from virtual primary care systems across five nations. PLOS Digit Health. 2023;2(11):e0000382. doi: 10.1371/journal.pdig.0000382 37939131 PMC10631700

[20] Kruk ME, Gage AD, Arsenault C, Jordan K, Leslie HH, Roder-DeWan S, et al. High-quality health systems in the Sustainable Development Goals era: Time for a revolution. Lancet Glob Health. 2018;6(11):e1196–252. doi: 10.1016/S2214-109X(18)30386-3 30196093 PMC7734391

[21] Taylor-Ide D, Taylor CE. Ding Xian: the first example of community-based development. In: Taylor-Ide D, Taylor CE, editors. Just and lasting change: when communities own their futures. Baltimore and London: Johns Hopkins University Press; 2002. p. 93–101.

[22] Newell KW. Health by the People. Geneva, Switzerland: World Health Organization; 1975. https://iris.who.int/items/0fafe19d-cf69-44c8-902b-6b83c97a52d9

[23] Werner D. The village health worker: lackey or liberator? World Health Forum. 1981;2(1):46–68. 10236800

[24] Sidel VW. The barefoot doctors of the People’s Republic of China. N Engl J Med. 1972;286(24):1292–300. doi: 10.1056/NEJM197206152862404 4401952

[25] Sidel V, Sidel R. The health care delivery system of the People’s Republic of China. In: Newell K, editor. Health by the People. Geneva, Switzerland: World Health Organization; 1975. pp. 1–12.

[26] Fang X. Barefoot Doctors and Western Medicine in China. Rochester, New York, USA: University of Rochester Press; 2012.

[27] Fang X. Taking off or putting on shoes?: Barefoot doctors, mental-manual labor, and the health revolution in Mao’s China. World Dev. 2025;194:107009. doi: 10.1016/j.worlddev.2025.107009

[28] Perry H. Health for the People: National Community Health Programs from Afghanistan to Zimbabwe. Baltimore, MD, USA: USAID/Jhpiego; 2020. https://chwcentral.org/wp-content/uploads/2021/11/Health_for_the_People_Natl_Case%20Studies_Oct2021.pdf

[29] Knowles M, Crowley AP, Vasan A, Kangovi S. Community health worker integration with and effectiveness in health care and public health in the United States. Annu Rev Public Health. 2023;44:363–81. doi: 10.1146/annurev-publhealth-071521-031648 37010928

[30] Chowdhury AMR, Ahmed Y, Islam K. 50 Years of Bangladesh Advances in Health. Dhaka, Bangladesh: Bangladesh Health Watch and University Press Limited; 2023.

[31] Scott K, Glandon D, Adhikari B, Ummer O, Javadi D, Gergen J. India’s auxiliary nurse-midwife, anganwadi worker, and accredited social health activist programs. In: Perry H, editor. Health for the people: National community health programs from Afghanistan to Zimbabwe. Baltimore, MD, USA: USAID/Jhpiego; 2020. pp. 113–33.

[32] Damtew Z, Abrehm S, Zulliger R, Moges A, Teklu A, Perry H. Ethiopia’s health extension program. In: Perry H, editor. National community health programs: Descriptions from Afghanistan to Zimbabwe. Baltimore, MD, USA: USAID/Jhpiego; 2020. pp. 75–82.

[33] Scott K, Beckham S, Gross M, Pariyo G, Rao K, Cometto G. What do we know about community health workers? A systematic review of existing reviews. Geneva: World Health Organization; 2017. 10.1186/s12960-018-0304-x PMC6097220 30115074

[34] WHO. WHO guideline on health policy and system support to optimize community health worker programmes Geneva: World Health Organization; 2018. Available from: https://apps.who.int/iris/bitstream/handle/10665/275474/9789241550369-eng.pdf?ua=1 30431747

[35] Chou VB, Friberg IK, Christian M, Walker N, Perry HB. Expanding the population coverage of evidence-based interventions with community health workers to save the lives of mothers and children: An analysis of potential global impact using the Lives Saved Tool (LiST). J Glob Health. 2017;7(2):020401. doi: 10.7189/jogh.07.020401 28959436 PMC5592116

[36] Schleiff M, Kumapley R, Freeman PA, Gupta S, Rassekh BM, Perry HB. Comprehensive review of the evidence regarding the effectiveness of community-based primary health care in improving maternal, neonatal and child health: 5. equity effects for neonates and children. J Glob Health. 2017;7(1):010905. doi: 10.7189/jogh.07.010905 28685043 PMC5491949

[37] Prost A, Colbourn T, Seward N, Azad K, Coomarasamy A, Copas A, et al. Women’s groups practising participatory learning and action to improve maternal and newborn health in low-resource settings: A systematic review and meta-analysis. Lancet. 2013;381(9879):1736–46. doi: 10.1016/S0140-6736(13)60685-6 23683640 PMC3797417

[38] Perry H, Morrow M, Davis T, Borger S, Weiss J, DeCoster M, et al. Care Groups II: A summary of the child survival outcomes achieved using volunteer community health workers in resource-constrained settings. Glob Health Sci Pract. 2015;3(3):370–81. doi: 10.9745/GHSP-D-15-00052 26374799 PMC4570012

[39] Perry H, de Broucker G, Ozawa S, Inagaki Y, Tam Y, Dyson J. The Investment Case for Community-based Primary Health Care and Community Health Workers in Low-Income and Lower Middle-Income Countries. New York, New York, USA: UNICEF; 2025.

[40] Ballard M, Johnson A, Mwanza I, Ngwira H, Schechter J, Odera M. Community health workers in pandemics: Evidence and investment implications. Glob Health Sci Pract. 2022;10(2). 10.9745/GHSP-D-21-00648 PMC9053152 35487542

[41] Frieden TR, Jaffe MG. Saving 100 million lives by improving global treatment of hypertension and reducing cardiovascular disease risk factors. J Clin Hypertens (Greenwich). 2018;20(2):208–11. doi: 10.1111/jch.13195 29370471 PMC8031351

[42] Schutte AE, Srinivasapura Venkateshmurthy N, Mohan S, Prabhakaran D. Hypertension in low- and middle-income countries. Circul Res. 2021;128(7):808–26. 10.1161/CIRCRESAHA.120.318729 PMC8091106 33793340

[43] Sun Y, Mu J, Wang DW, Ouyang N, Xing L, Guo X, et al. A village doctor-led multifaceted intervention for blood pressure control in rural China: An open, cluster randomised trial. Lancet. 2022;399(10339):1964–75. doi: 10.1016/S0140-6736(22)00325-7 35500594

[44] Mishra SR, Neupane D, Preen D, Kallestrup P, Perry HB. Mitigation of non-communicable diseases in developing countries with community health workers. Glob Health. 2015;11:43. doi: 10.1186/s12992-015-0129-5 26555199 PMC4641398

[45] Yeboah B. Primary Health Care Transformation in Africa: Leveraging the 2 Million CHW Acceleration Framework to Pave the Way for Equitable Health Systems in Africa. International Conference on Primary Health Care. Addis Ababa, Ethiopia; 2025.

[46] Olaniran A, Banke-Thomas A, Bar-Zeev S, Madaj B. Not knowing enough, not having enough, not feeling wanted: Challenges of community health workers providing maternal and newborn services in Africa and Asia. PLoS One. 2022;17(9):e0274110. doi: 10.1371/journal.pone.0274110 36083978 PMC9462785

[47] Berman PA, Gwatkin DR, Burger SE. Community-based health workers: Head start or false start towards health for all?. Soc Sci Med. 1987;25(5):443–59. doi: 10.1016/0277-9536(87)90168-7 3118477

[48] d’Harcourt E, Chowdhury M, Perry H. Coerced volunteers or community leaders? The complex ethics of community health worker payments. Glob Health: Sci Pract. 2026 (in press).

[49] Buchan J, Couper ID, Tangcharoensathien V, Thepannya K, Jaskiewicz W, Perfilieva G, et al. Early implementation of WHO recommendations for the retention of health workers in remote and rural areas. Bull World Health Organ. 2013;91(11):834–40. doi: 10.2471/BLT.13.119008 24347707 PMC3853959

[50] Tanser F, Gijsbertsen B, Herbst K. Modelling and understanding primary health care accessibility and utilization in rural South Africa: An exploration using a geographical information system. Soc Sci Med. 2006;63(3):691–705. doi: 10.1016/j.socscimed.2006.01.015 16574290

[51] Giugliani C, Zulliger R. The Community Health Agent Program of Brazil. In: Perry H, editor. Health for the People: National Community Health Programs from Afghanistan to Zimbabwe. Baltimore, MD, USA: USAID/Jhpiego; 2020. pp. 61–73.

[52] Lu C, Palazuelos D, Luan Y, Sachs SE, Mitnick CD, Rhatigan J, et al. Development assistance for community health workers in 114 low- and middle-income countries, 2007-2017. Bull World Health Organ. 2020;98(1):30–9. 10.2471/BLT.19.235499 PMC6933433 31902960

[53] Apeagyei AE, Bisignano C, Elliott H, Hay SI, Lidral-Porter B, Nam S, et al. Tracking development assistance for health, 1990-2030: historical trends, recent cuts, and outlook. Lancet. 2025;406(10501):337–48. doi: 10.1016/S0140-6736(25)01240-1 PMC12439094 40680759

[54] Hanson K, Brikci N, Erlangga D, Alebachew A, De Allegri M, Balabanova D, et al. The Lancet Global Health Commission on financing primary health care: Putting people at the centre. Lancet Glob Health. 2022;10(5):e715–72. doi: 10.1016/S2214-109X(22)00005-5 35390342 PMC9005653

[55] Frankel S. The community health worker: effective programmes for developing countries. Oxford, England: Oxford University Press; 1992.

[56] SIPRI. World military expenditure passes $2 trillion for first time: Stockholm International Peace Research Institute; 2022. Available from: https://www.sipri.org/media/press-release/2022/world-military-expenditure-passes-2-trillion-first-time

[57] Sachs J, Perry HB. Needed: A financing breakthrough at the UN High-level Meeting on Universal Health Coverage. Lancet. 2023;402(10411):1403–4. doi: 10.1016/S0140-6736(23)01924-4 37734397

[58] Fendall NR. Auxiliaries and primary medical care. Bull N Y Acad Med. 1972;48(10):1291–300. 4508857 PMC1806876

